# Prevalence and patterns of dental needle tip deformation during maxillary buccal and palatal infiltration anesthesia

**DOI:** 10.3389/froh.2026.1774756

**Published:** 2026-06-12

**Authors:** Mohammed Amjed Alsaegh, Taibah Abdullah Alkandari, Mariam Mohammad Jumah, Zahraa Bader Khasroh, Farah Ahmad Alenezi

**Affiliations:** 1 Department of Oral and Craniofacial Health Sciences, College of Dental Medicine, University of Sharjah. Sharjah, United Arab Emirates; 2 Research Institute for Medical and Health Sciences, University of Sharjah, Sharjah, United Arab Emirates; 3 University Dental Hospital Sharjah, University of Sharjah, Sharjah, United Arab Emirates

**Keywords:** bevel, dental interns, dental needles, dental students, infiltration anesthesia, local anesthesia, needle deformation

## Abstract

**Objectives:**

Needle tip deformation is a recognized occurrence during dental local anesthesia that may affect injection safety and efficacy. This study aimed to assess the prevalence, extent, and direction of needle tip deformation and its association with operator level, bone contact sensation, and injection approach in maxillary infiltration.

**Methods:**

This cross-sectional study analyzed 180 dental needle samples, including 90 experimental and 90 control samples. The experimental group was subdivided into three injection approaches: buccal infiltration only, palatal infiltration only, and sequential buccal-then-palatal infiltration performed using the same needle. Needle tip deformation was examined using a field-emission scanning electron microscope (FE-SEM), and the extent of deformation was quantified with ImageJ software. Statistical analyses compared the prevalence, extent, and direction of deformation across practitioner levels and injection approaches.

**Results:**

Needle tip deformation occurred more frequently in the experimental group (55.6%) compared with controls (1.1%) (*χ*^2^ = 65.691, *p* < .001). No significant differences in the prevalence of deformation were observed across injection approaches (*χ*^2^ = 2.520, *p* = .284). Fourth-year dental students and interns demonstrated comparable rates of deformation (53.3% vs. 57.8%) (*χ*^2^ = 0.180, *p* = .671) and non-different extent of deformation (t = 0.588, *p* = .588). The direction of deformation (inward 52.2% vs. outward 47.8%) showed no significant association with operator level (*χ*^2^ = 6.523, *p* = .163) and no significant difference in the extent of deformation (t = −1.275, *p* = .209). Although the sensation of bone contact varied significantly by injection type (*χ*^2^ = 13.289, *p* = .004), it was not associated with actual needle tip deformation (*ρ* = 0.140, *p* = 0.189).

**Conclusion:**

Needle tip deformation occurred in more than half of maxillary infiltration procedures. The prevalence and extent of deformation were not influenced by operator level, bone contact, or the infiltration approach in buccal, palatal, and sequential buccal-palatal injections. Future studies should assess patient-centered and anesthetic outcomes to determine its clinical relevance.

## Introduction

Local anesthesia is the administration of an anesthetic solution in close proximity to a nerve trunk or terminal nerve endings, resulting in transient numbness and loss of sensation in the region innervated by that nerve through the blockade of nociceptive stimulation (1). In dentistry, local anesthesia can be achieved using several approaches, including infiltration (field block), regional nerve blocks, and a range of supplementary techniques (1).

In the maxillary arch, infiltration anesthesia is the most commonly used technique for localized anesthesia. Buccal infiltration is administered at the mucobuccal fold with the bevel oriented toward bone, whereas palatal infiltration is used mainly to anesthetize the adjacent soft tissues (2).

Dental anesthesia needles should be designed with a bevel that enables smooth penetration of soft tissues while reducing the risk of vascular or neural injury (3). Variations in bevel design, such as the standard and surgical bevels, influence the extent of deformation. Surgical bevel needles are typically less susceptible to deformation because their shorter bevel length and dual-edged sharpening provide greater structural stability during use (4). Needle characteristics also play a role in deformation; for instance, needles with larger inner diameters have been shown to carry a higher risk of deformation (5).

The type of dental procedure also influences the extent of needle deformation. Intraligamentary and intraseptal anesthesia often result in needle tip bending at angles ranging from 15° to 180°, frequently necessitating the use of multiple needles for multi-rooted teeth (4). Repeated use of needles, particularly during procedures such as inferior alveolar nerve blocks, has likewise been shown to increase deformation irrespective of bevel orientation (6). Deformed or barbed needles have been associated with greater soft-tissue trauma and pain. A randomized controlled trial have demonstrated that distorted tips may develop barbed hooks that exacerbate tissue injury, whereas bevel designs engineered to resist deformation can mitigate injection-related discomfort (7). Furthermore, the risk of nerve injury increases during withdrawal or reuse following bone contact, as barbed tips may damage nerve fascicles, including those of the lingual nerve, leading to complications such as paresthesia and trismus (3).

Local anesthesia training is a core component of dental education and progresses from theoretical teaching to supervised clinical practice, including simulation-based training (8). Differences between students and interns may reflect variation in clinical exposure and technical performance. One previous study reported a higher rate of inferior alveolar nerve block failure among dental students than interns (9). The present study examined whether such differences also extend to needle tip deformation during infiltration anesthesia.

Needle tip deformation has been reported at widely varying rates across different injection techniques. Direct inferior alveolar nerve blocks performed by dental students showed deformation in 50% of needles (10). Another investigation on the same technique documented deformation in almost 100% of samples (6). Infiltration anesthesia studies reported deformation in roughly 97% of needles when various bevel designs were used (7). Intraligamentary injections demonstrated a similarly high prevalence of 97.5% (11). Interestingly, although needles with barbed hooks exhibit higher withdrawal forces, this did not translate into greater pain perception, likely due to the overriding effect of local anesthesia (7).

To date, no study has investigated needle tip deformation during dental anesthesia in a comparative evaluation of dental students and interns. In addition, a gap persists in the literature regarding the prevalence of needle tip deformation associated with maxillary dental infiltration. Furthermore, no consensus exists on whether a single needle should be used for both buccal and palatal injections, or on the optimal sequence of administration when both sites are required. The present study was therefore conducted to evaluate the prevalence, extent, and direction of needle tip deformation following maxillary buccal and palatal infiltration anesthesia performed by fourth-year dental students and dental interns, and to examine whether deformation is affected by the injection approach in buccal, palatal, or sequential buccal–palatal injections or by operator level.

## Materials and methods

This cross-sectional study included the collection of needle samples from undergraduate fourth-year dental students and dental interns following maxillary infiltration anesthesia administered during maxillary tooth extractions at the University Dental Hospital Sharjah (UDHS). Ethical approval was obtained from the Research Ethics Committee of the University of Sharjah (Ref. no. REC-23-03-23-01-S).

The sample size was calculated using G*Power software (version 3.1.9.7; University of Düsseldorf, Germany). Based on the parameters reported by Skapetis et al. (6), an effect size of 0.4, a statistical power of 85%, and an alpha error of 5% were applied, yielding a required sample of 90 patients for the experimental group.

Informed consent was obtained from all patients and practitioners, including dental students and interns, after they were provided with detailed information sheets describing the study objectives and procedures. For participants younger than 16 years, informed consent was secured from their legal guardian prior to enrollment. All procedures were performed in accordance with established guidelines for submucosal infiltration anesthesia (2). For the buccal infiltration, the injection was delivered at the mucobuccal fold above the tooth apex with the bevel toward bone, the needle inserted 2 to 3 mm at a slight angulation, and approximately 0.6 to 1 mL of 2% lidocaine with 1:100,000 epinephrine deposited slowly over 30 s. For the palatal infiltration, the needle was inserted into the attached gingiva 5 to 10 mm from the free gingival margin with the bevel against the tissue. A small amount of anesthetic was deposited during penetration, and the needle was advanced until gentle bone contact was achieved, after which 0.2 to 0.3 mL of solution was administered. All injections were performed under direct supervision of a clinical instructor, who ensured consistent adherence to the standardized procedural protocol.

The inclusion criteria consisted of patients scheduled for maxillary tooth extraction at UDHS who were to receive both buccal and palatal infiltration anesthesia and were treated by either fourth-year dental students or dental interns. Eligible patients were required to be at least 15 years of age. Needles mishandled or used for more than the intended number of insertions were excluded; buccal-only and palatal-only injections each involved a single insertion, whereas the sequential buccal-then-palatal infiltration approach involved two controlled insertions.

All needles used in this study were identical, consisting of 27-gauge, 25-mm length, tri-beveled disposable dental needles from a single manufacturer (Denject, S. Korea). Each participant, whether a fourth-year dental student or a dental intern, administered both buccal and palatal infiltration anesthesia with the bevel oriented toward the bone. To ensure safety, researchers wore needlestick-resistant gloves during needle collection. After each injection, the needle was carefully detached from the syringe using Spencer Wells forceps, grasped near the hub to avoid tip contact, and then bent until fracture occurred at the hub, leaving the bevel tip intact.

For each needle, details regarding the injection approach, operator academic level, and any reported sensation of bone contact were documented. Used needles were stored in labeled test tubes according to injection category and practitioner level. Each tube contained 10 mL of 70% ethanol, which served as a storage medium for the collected samples.

The collected needle samples were categorized as buccal infiltration only, palatal infiltration only, or sequential buccal-then-palatal infiltration using the same needle. Each category was further subdivided by operator level (fourth-year dental student or intern). In total, 180 samples were included, comprising 90 unused controls and 90 experimental samples. The unused control needles were obtained from sealed packages to provide a baseline for bevel morphology, identify possible manufacturing irregularities, and confirm that any observed deformation was related to clinical use rather than handling or production defects. The experimental group was equally divided into six subgroups (*n* = 15 each): buccal, palatal, and sequential buccal-then-palatal infiltration using the same needle performed by either interns or fourth-year students. The control group consisted of 90 unused, sterile needles taken directly from sealed packages to provide baseline bevel morphology and detect any manufacturing irregularities. They were handled and imaged using the same protocol as experimental needles, ensuring that observed deformation reflected clinical use rather than handling or production defects.

To minimize procedural and observer bias, injection approaches were allocated according to a predetermined schedule to ensure balanced distribution between fourth year students and interns. For all clinical procedures, the sequence of injections was standardized, with the buccal infiltration always administered before the palatal infiltration when both sites were required. In addition, the examiner responsible for field-emission scanning electron microscope (FE-SEM) imaging and quantitative analysis was blinded to both the operator level and the injection approach of each needle sample.

All dental needles were examined under ×8 magnification using a surgical operating microscope (A-6, Global Surgical Cooperation, USA) to verify bevel orientation to the left side, ensuring lateral visualization of the needle tip. For FE-SEM analysis, all collected needles, including controls, were mounted on aluminum stubs with care taken to avoid direct tip contact and positioned in a standardized orientation. A field-emission scanning electron microscope (FE-SEM, Apreo 2, ThermoFisher Scientific, Massachusetts, USA) was used at ×100 and ×500 magnifications for imaging.

**Figure 1:**
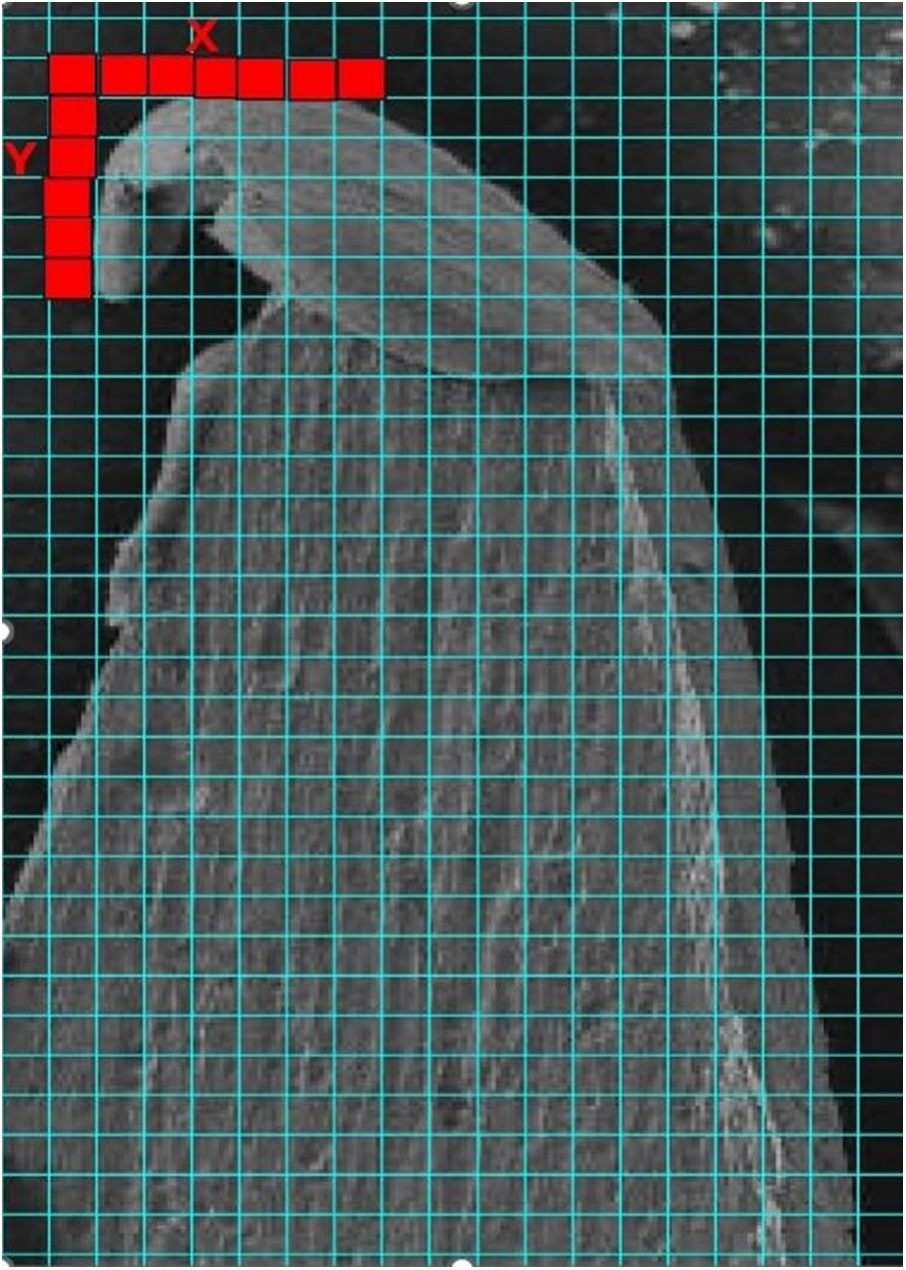
Quantitative measurement of dental needle tip deformation. Scanning electron micrograph (500×) of a deformed needle tip overlaid with a 5 × 5 µm grid using ImageJ software, with arbitrary units represented by box numbers.

**Figure 2:**
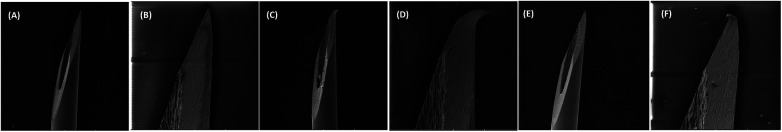
Field-emission scanning electron micrographs of dental needle tips at 100× and 500× magnification. **(A, B)** Intact needle tips at 100× **(A)** and 500× **(B) (C, D)** Outwardly deformed needle tips at 100× **(C)** and 500× **(D) (E, F)** Inwardly deformed needle tips at 100× **(E)** and 500× **(F).**

Images were acquired and analyzed using ImageJ software (version 1.53t; NIH, Bethesda, MD, USA). To quantify needle tip deformation, images were aligned on 5.5-µm grid boxes, and vertical and horizontal deviations of the needle tip were measured in arbitrary units (box numbers) (Figure 1). Based on the long axis, deformation direction was classified as outward (away from the lumen) or inward (toward the lumen) (Figure 2).

All statistical analyses were performed using SPSS software, version 28.0 for Macintosh (IBM Corp., Chicago, IL, USA). Categorical variables were summarized as frequencies and percentages, while continuous variables were reported as minimum, maximum, and mean values. Chi-square tests were applied to assess differences in categorical variables, Student's t-tests were used to compare the extent of needle tip deformation, and two-way ANOVA was employed to examine the effect of operator training level across injection approaches. Spearman's correlation coefficient was applied to evaluate associations between ordinal and continuous variables. A *p*-value ≤ 0.05 was considered statistically significant.

## Results

A total of 180 needles were analyzed, comprising 90 experimental samples (50%) and 90 controls (50%). Among the experimental group, 74 needles (82.2%) were obtained from male patients and 16 (17.8%) from female patients, with ages ranging from 15 to 65 years and a mean age of (37.22 ± 11.90) years. Of the 90 experimental needles, 45 were used by interns and 45 by fourth-year dental students. Each group of 45 was further subdivided equally into three subgroups: buccal infiltration only (*n* = 15), palatal infiltration only (*n* = 15), and sequential buccal-then-palatal infiltration using the same needle (*n* = 15) (Table 1).

**Table 1:** Demographic and sample characteristics of the study population.

Groups	Experimental	Control
Number	*N* = 90 (50%)	*N* = 90 (50%)
Gender	Males = 74 (82.3%) Females = 16 (17.7%)	
Age	37.22 ± 11.904	
Operator level	Fourth-year dental students: *N* = 45 Dental interns: *N* = 45	
Site of injection	Anterior: *N* = 12 (13.3%), Premolars: *N* = 33 (36.7%), Molars: *N* = 45 (50.0%)	
Injection approaches	Buccal: *N* = 30 (15 Fourth-year dental students and 15 dental interns) Palatal: *N* = 30 (15 Fourth-year dental students and 15 dental interns) Buccal-palatal: *N* = 30 (15 Fourth-year dental students and 15 dental interns)	

Group comparability and associations with needle deformation are summarized in Table 2. Fourth-year dental students and dental interns were comparable with respect to tooth type, patient gender, and patient age. Likewise, the buccal, palatal, and buccal-palatal injection approach groups did not differ significantly in these variables, confirming comparability across groups. In addition, needle deformation was not significantly associated with patient gender, tooth location, or patient age.

**Table 2:** Group comparability and association of selected variables with needle deformation.

Comparison	Variable	Test statistic	*p* value
Operator level: fourth-year dental students vs. dental interns	Tooth type	*χ*^2^ = 20.955	.103
Operator level: fourth-year dental students vs. dental interns	Patient gender	χ^2^ = 1.216	.270
Operator level: fourth-year dental students vs. dental interns	Patient age	t = −0.441	.660
Injection approach: buccal vs. palatal vs. buccal-palatal	Tooth type	χ^2^ = 17.773	.932
Injection approach: buccal vs. palatal vs. buccal-palatal	Patient gender	χ^2^ = 1.976	.372
Injection approach: buccal vs. palatal vs. buccal-palatal	Patient age	F = 1.491	.231
Association with needle deformation	Patient gender	χ^2^ = 0.380	.538
Association with needle deformation	Tooth location	χ^2^ = 1.720	.423
Association with needle deformation	Patient age	χ^2^ = 6.566	.255

Regardless of practitioner level, needle tip deformation occurred in 50 experimental cases (55.6%) compared with 1 control case (1.1%). The chi-square test demonstrated a significantly higher rate of deformation in the experimental group than in the controls (*χ*^2^ = 65.691, *p* < .001).

**Figure 3:**
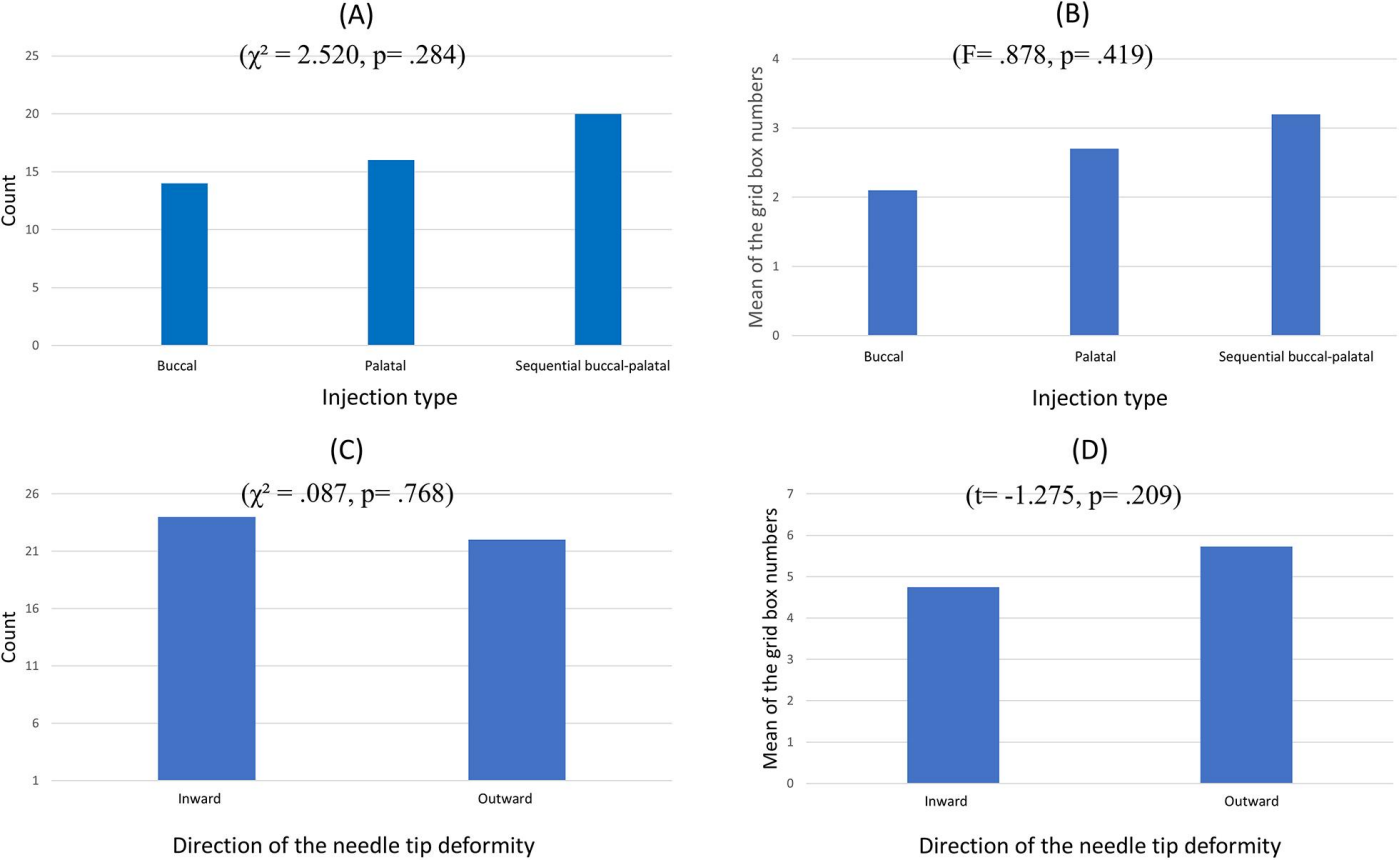
Prevalence, extent, and direction of needle tip deformation. **(A)** Prevalence of needle tip deformation across buccal, palatal, and sequential buccal-palatal infiltration. **(B)** Extent of needle tip deformation across injection approaches, expressed as grid box numbers. **(C)** Prevalence of inward and outward needle tip deformation in the experimental group. **(D)** Extent of inward and outward needle tip deformation in the experimental group, expressed as grid box numbers.

No significant differences in needle deformation were observed across injection approaches among all participants (*χ*^2^ = 2.520, *p* = .284), nor within the fourth-year student group (*χ*^2^ = 1.607, *p* = .448) or the intern group (*χ*^2^ = 1.275, *p* = .529). Likewise, the extent of deformation did not differ significantly among the three injection approaches (F = 0.878, *p* = .419) (Figure 3).

**Figure 4:**
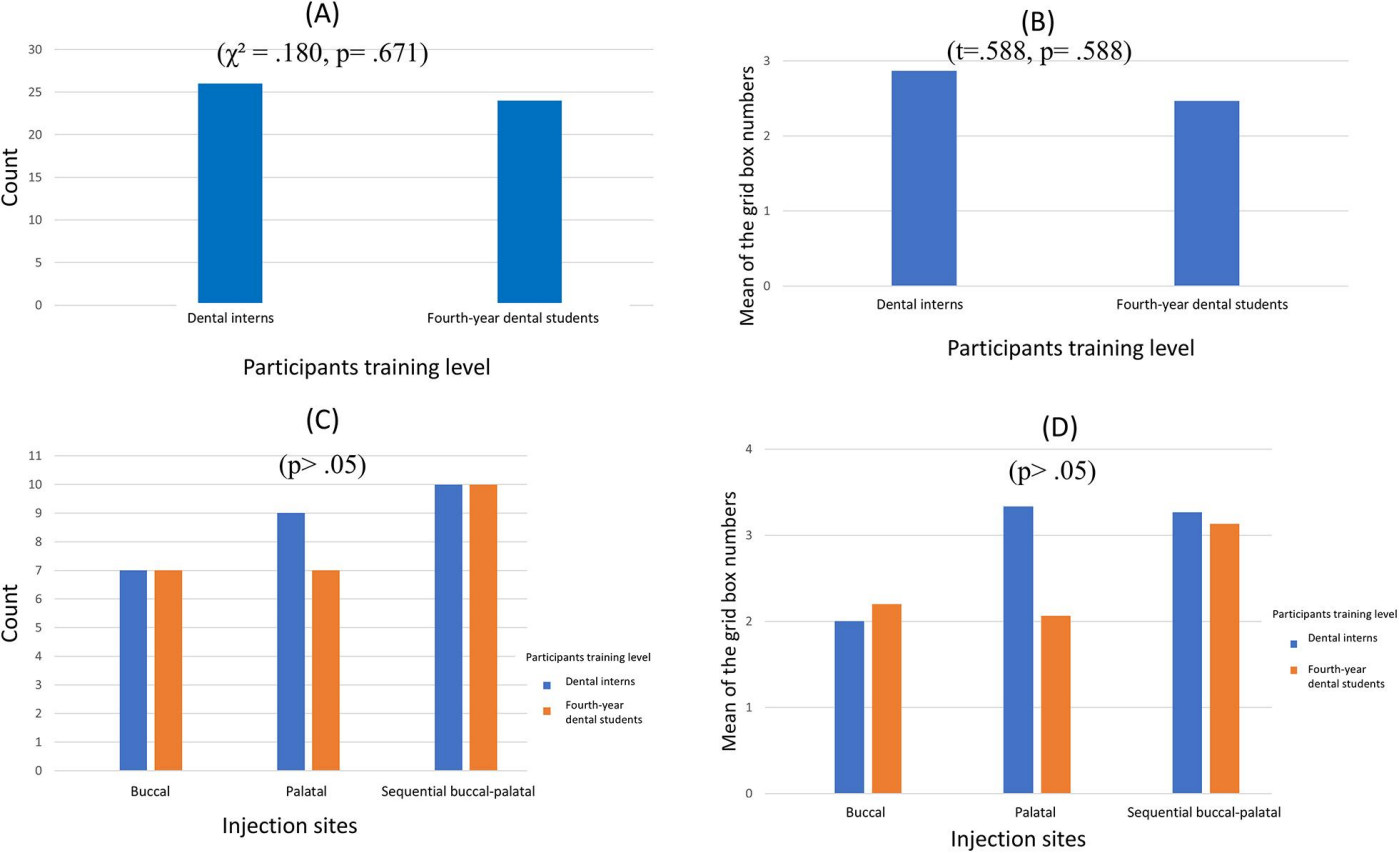
Prevalence and extent of needle tip deformation between fourth-year dental students and interns. **(A)** Prevalence of deformation by practitioner level. **(B)** Extent of deformation by practitioner level measured in grid box numbers. **(C)** Prevalence of deformation by injection approach within practitioner levels. **(D)** Extent of deformation by injection approach within practitioner levels measured in grid box numbers.

Needle tip deformation was observed in 24 cases (53.3%) among fourth-year dental students and in 26 cases (57.8%) among interns. No statistically significant difference in overall deformation occurrence was found between the two groups (*χ*^2^ = 0.180, *p* = .671) (Figure 4). Likewise, Student's t-test indicated no significant difference in the extent of deformation between students and interns (t = 0.588, *p* = .588) (Figure 4).

Within the fourth-year student group, deformation occurred in 53.3% of samples [buccal approach: 7 [46.6%], palatal approach: 7 [46.6%], buccal-palatal approach: 10 [66.6%]]. Among interns, deformation was present in 57.7% of samples [buccal approach: 7 [46.6%], palatal approach: 9 [60.0%], buccal-palatal approach: 10 [66.6%]]. Two-way ANOVA revealed no significant effect of participant level (F = 0.340, *p* = .561), injection approach (F = 0.860, *p* = .427), or their interaction (F = 0.419, *p* = .659) on the extent of deformation (Figure 4).

Chi-square analysis further confirmed no significant differences in deformation rates between students and interns at individual injection approaches: buccal (*χ*^2^ = 0.000, *p* = 1.000), palatal (*χ*^2^ = 0.536, *p* = .464), and buccal-palatal (*χ*^2^ = 0.000, *p* = 1.000). The overall difference across all approaches was also non-significant (*χ*^2^ = 0.180, *p* = .671) (Figure 4).

Needle tip deformation was observed in 46 experimental samples, with 24 (52.2%) classified as inward and 22 (47.8%) as outward. In the control group, a single case of needle tip deformation (1.1%) was detected, which was inward. The distribution of deformation direction was not statistically significant (*χ*^2^ = 0.087, *p* = .768). No significant differences in deformation direction were found between participant levels (*χ*^2^ = 6.523, *p* = .163) (Figure 3). Likewise, the extent of deformation did not differ significantly between inward and outward deformations in the experimental group (t = –1.275, *p* = .209) (Figure 3).

Bone contact was not reported in 12 cases, noted only on the buccal side in 19 cases (18 during buccal infiltrations and 1 during buccal-palatal infiltrations), only on the palatal side in 37 cases (30 during palatal infiltrations and 7 during buccal-palatal infiltrations), and on both buccal and palatal sides in 22 buccal-palatal infiltrations (*χ*^2^ = 13.289, *p* = .004). The Spearman correlation coefficient demonstrated no significant association between the operator's perceived sensation of bone contact and the actual occurrence of needle deformation across all injection approaches (*ρ* = 0.140, *p* = 0.189).

## Discussion

This study demonstrated that needle tip deformation is a frequent occurrence during dental infiltration procedures, with no significant association with operator level, injection approach, or perceived bone contact. Inward and outward deformations occurred at nearly comparable frequencies. These findings are clinically relevant because needle deformation may increase tissue trauma and procedural complications.

In the present study, needle tip deformation was observed in 1.1% of control needles and 55.6% of experimental needles after maxillary infiltration anesthesia. Comparable findings have been reported, with one investigation documenting 50% deformation following direct inferior alveolar nerve blocks administered by dental students (10). However, another study on inferior alveolar nerve block reported deformation in nearly all needles examined (100%) (6). A SEM-based study identified deformation in approximately 97% of needles used for infiltration anesthesia with variable bevel types (7). Intraligamentary injections demonstrated a prevalence of 97.5% (11). Other research has highlighted differences in deformation rates across injection approaches, reporting 54.5% in truncal blocks and 57.9% in buccal infiltrations (3). Such variations across studies may be attributed to differences in injection technique, needle length and gauge, internal core diameter, manufacturer, and bevel design. Notably, one study demonstrated that scalpel-designed bevel needles exhibited a higher frequency of barbs compared with triple-bevel and regular-bevel designs (7). Earlier investigations further showed that needles with the same gauge and length but different internal diameters exhibited varying degrees of bevel deformation, underscoring the influence of design features beyond gauge (3, 5). Moreover, repeated use of the same needle has been shown to exacerbate deformation, thereby increasing the potential for complications during local anesthesia (7).

A bent or deformed needle may contribute to tissue trauma during withdrawal or repeated insertion, potentially leading to greater patient discomfort or postoperative soreness. The high deformation rate observed in this study underscores the need for clinicians to remain vigilant, inspect needles carefully, and avoid repeated use across multiple injections. Although the present study did not directly evaluate anesthetic efficacy, pain perception, or complication rates, the findings highlight the importance of further research to determine whether needle deformation has a measurable impact on these clinical outcomes.

With respect to unused needles, previous investigations have reported findings comparable to the present study, noting that 12%–38% exhibited manufacturing defects or production-related irregularities (5). Even unused needles may exhibit minor bevel imperfections related to manufacturing, packaging, or handling. Because the needles used in this study were tri-beveled, subtle pre-existing irregularities cannot be completely excluded, although their frequency was very low in the control group.

Although the differences were not statistically significant, a trend was observed toward greater deformation from buccal to palatal to buccal-palatal injections. This may suggest that, when both injections are required, administering the buccal injection first could be advantageous, although this remains hypothetical. The slightly higher deformation during palatal injections may relate to the tautness of palatal tissues and the short distance to bone contact. These findings also support considering a new needle for each injection approach to reduce tissue trauma and possible anesthetic leakage.

Our findings indicated that neither the prevalence nor the extent of needle tip deformation differed significantly between fourth-year dental students and intern dentists. However, this result should be interpreted within the limits of the study, as these groups represent adjacent training stages with only a modest difference in clinical experience. A previous study similarly found no significant association between bevel deformation and the surgical experience of dentistry graduates or oral surgery residents when performing truncal block and infiltration techniques (3). In contrast, another investigation reported that needle tip deformation following direct inferior alveolar nerve block was greater among staff dentists compared with dental students (10).

Standardization in training may contribute to comparable outcomes in terms of needle deformation between dental students and interns. This may also be attributed to the recent integration of simulation-based techniques into the preclinical teaching of dental students at the University of Sharjah. Evidence from a recent quasi-experimental study at the University of Minnesota, which compared student-to-student practice with manikin-based simulation among dental hygiene students, supports this approach. The simulation group achieved significantly higher skill levels, although self-confidence did not differ notably between the groups (12). In contrast, another study reported that success rates achieved on manikin models do not necessarily translate to equivalent performance *in vivo*. While simulation models serve as valuable didactic tools, training should continue to emphasize real-life clinical experience. In addition, students should receive chairside feedback using established evaluation methods (8).

The clinical significance of bevel deformation direction has been highlighted by Stacy et al., who suggested that outward twisting of the bevel upon withdrawal may cause greater trauma to the neurovascular bundle of the inferior alveolar and lingual nerves compared with inward distortion (10). This observation supports the recommendation of orienting the bevel toward the bone when administering an inferior alveolar nerve block (10). In the present study, inward and outward needle tip deformations occurred at nearly equal frequencies, with no statistically significant differences observed between experimental groups. These findings indicate that bevel orientation does not appear to influence either the direction or the extent of deformation. A previous study similarly reported no correlation between bevel orientation and deformation direction (6). Consistent with our results, Dau et al. also observed no significant differences in needle tip distortion patterns following infiltration anesthesia (7).

Interestingly, other investigations have suggested that bevel orientation at the time of bone contact may influence the direction of deformation. When the bevel faces the bone surface, inward deflection is more likely, whereas a medial orientation favors outward distortion (10). In that study, when local anesthesia was performed by dental students, 64% of deformed needle tips were inwardly barbed and 36% outwardly, whereas procedures performed by staff showed 37.1% inward barbs and 62.9% outward (10).

No significant correlation was found between perceived bone contact and actual needle tip deformation. This may partly reflect the subjective nature of tactile perception during injection. Previous studies have reported inconsistent findings, with some showing no association between bone contact and deformation and others reporting greater deformation after bone contact (3, 13). These discrepancies indicate that the effect of bone contact on needle deformation remains uncertain and warrants further study.

A limitation of the present study is that pre-existing deformation in the same clinically used needles could not be assessed directly because of sterility considerations. Although one unused control needle showed deformity, the very low frequency in controls compared with experimental samples suggests that most deformities developed after clinical use. The study was also limited to one needle brand and one bevel design, and patient-reported outcomes such as injection pain and postoperative discomfort were not evaluated. Future studies should include multiple manufacturers, different bevel designs, and validated patient-reported outcomes.

## Conclusions

Needle tip deformation was observed in more than half of maxillary infiltration procedures performed by both fourth-year dental students and interns. The prevalence and extent of deformation were not significantly influenced by operator level, perceived bone contact, or the infiltration approach used for buccal, palatal, and sequential buccal-palatal injections. Future studies should incorporate validated patient-centered and anesthetic outcome measures to clarify the clinical relevance of needle tip deformation.

## Data Availability

The raw data supporting the conclusions of this article will be made available by the authors, without undue reservation.
